# Characterization of knockin mice at the *Rosa26*, *Tac1* and *Plekhg1* loci generated by homologous recombination in oocytes

**DOI:** 10.1371/journal.pone.0193129

**Published:** 2018-02-27

**Authors:** Youmei Wu, María José Luna, Lauren S. Bonilla, Nicholas J. P. Ryba, James M. Pickel

**Affiliations:** 1 National Institute of Dental and Craniofacial Research, National Institutes of Health, Bethesda, Maryland, United States of America; 2 National Institute of Mental Health, National Institutes of Health, Bethesda, Maryland, United States of America; Lewis Katz School of Medicine at Temple University, UNITED STATES

## Abstract

Design and engineering of complex knockin mice has revolutionized the *in vivo* manipulation of genetically defined cells. Recently development of the bacterial clustered regularly interspersed short palindromic repeats (CRISPR) associated protein 9 (Cas9) system for single site cleavage of mammalian genomes has opened the way for rapid generation of knockin mice by targeting homology directed repair to selected cleavage sites. We used this approach to generate new lines of mice that will be useful for a variety of aspects of neuroscience research. These lines have been bred to homozygosity and details of the expression and function of the transgenes are reported. Two lines target the *Rosa26*-locus and have been engineered to allow Cre-dependent expression of the avian tva receptor, and Cre-dependent expression of a cell surface targeted spaghetti-monster carrying many copies of the “ollas-tag”. Another line expresses red fluorescent protein and tva in *Tac1*-positive neurons; the fourth line targets FlpO expression to *Plekhg1* expressing neurons, providing a powerful approach to modify gene expression in thalamic excitatory neurons.

## Introduction

Nuclease based methods, including zinc-finger nucleases [1], transcription activator-like effector nucleases (TALENS, [2]) and most recently the bacterial clustered regularly interspaced short palindromic repeats (CRISPR)-associated protein 9 (Cas9) system [3–6] have become standard approaches for single-site cleavage of the mammalian genome. CRISPR, in particular, provides a simple, versatile and robust tool for generating gene knock-outs and targeted mutations in a variety of mammalian models [7, 8]. Using CRISPR, gene-knockout in mice or rats is now as straightforward and fast as producing transgenic animals and has extraordinary efficiency [9]. In addition, there are many examples where long single-stranded oligonucleotide mediated homologous recombination at CRISPR-directed double strand breaks occurs with reasonable fidelity and high efficiency, facilitating the engineering of knockin-animal models carrying short targeted sequences [9]. There have also been a number of reports where CRISPR-targeted approaches have been used to direct long-knockins of plasmid DNA using homologous recombination in zygotes [7, 10–12]. Here we report the generation of several lines of complex knockin mice using such CRISPR technology using a simple approach that works at all genomic loci that we have attempted to target.

Four of these lines have been bred to homozygosity and have been characterized for transgene expression and function. These lines of mice have been preserved as frozen sperm and will be available to the community on request.

## Materials and methods

### Ethics statement

This study was carried out in strict accordance with the recommendations in the Guide for the Care and Use of Laboratory Animals of the National Institutes of Health. Protocols were approved by the National Institute of Dental and Craniofacial Research Animal Care and Use Committee (protocols 14–743, 13–702 and 15–763) and the National Institute of Mental Health Animal Care and Use Committee (protocol TGC01). Mice were euthanized using CO_2_ inhalation followed by cervical dislocation. Briefly, mice were slowly exposed to 100% CO_2_ at a flow rate displacing 10–30% of the cage volume/minute, once mice were unconscious, death was confirmed by cervical dislocation. In addition, some experiments were performed using ketamine / xylazine anesthesia, and all efforts were made to minimize suffering.

### Animal husbandry

Mice were group housed in standard cages on ventilated racks (Techniplast, maximum 4 animals per cage or Allentown, maximum 5 animals per cage). All cages had standard wire tops and were provided with litter as well as a cardboard tube and nesting material for environmental enrichment. Mice received feed (Rodent chow: NIH-07) and deionized water *ad libitum*. Mice were kept in standard conditions (mean±SD room temperate: 22.5 ± 2°C in a 12:12 h light:dark cycle, lights off at 6 pm). Generation of knockin mice utilized singly housed stud and vasectomized males which were repeatedly mated with superovulated or normal females to generate fertilized oocytes and pseudopregnant recipients (~120 adult mice). 86 potential founder mice were born and genotyped; of these the 14 positive knockin mice were crossed to wild type animals; offspring were used for characterization of the lines. Once it was established that the lines for each construct had equivalent expression, 4 selected lines were chosen for sperm freezing, generation of homozygotes and the characterization reported here. In total these experiments involved an additional 278 mice.

### Generation of sgRNA, targeting vectors and mixtures for pronuclear injection

Potential Cas9 cleavage sites were identified by screening genomic regions of interest using online software [13] and the updated version http://chopchop.cbu.uib.no [14]. Predicted sequences were assessed first for proximity to the desired targeting site, second for high efficiency and third for lack of off target sequences. We were more willing to compromise and accept sgRNAs with several 3-base off-target sites, if these contained at least one difference in the twelve 3’ residues, than select a less efficient target or one that was further from the desired location. If a predicted sgRNAs started with a GA, Sp6 RNA polymerase was chosen for RNA synthesis; if the desired sgRNA started GG, T7 RNA polymerase was used. If other bases were present at the 5’-end, target sequences were modified by adding an extra G to the sgRNA or changing the starting nucleotide to a G or both to generate a GG or GA 5’-end (see S1 Table). The sgRNAs, were all tested and worked efficiently for directing in vitro cleavage of PCR-amplified targets by Cas9 (S1 Fig), and also in the generation of knockin mice (see Results).

**Fig 1:**
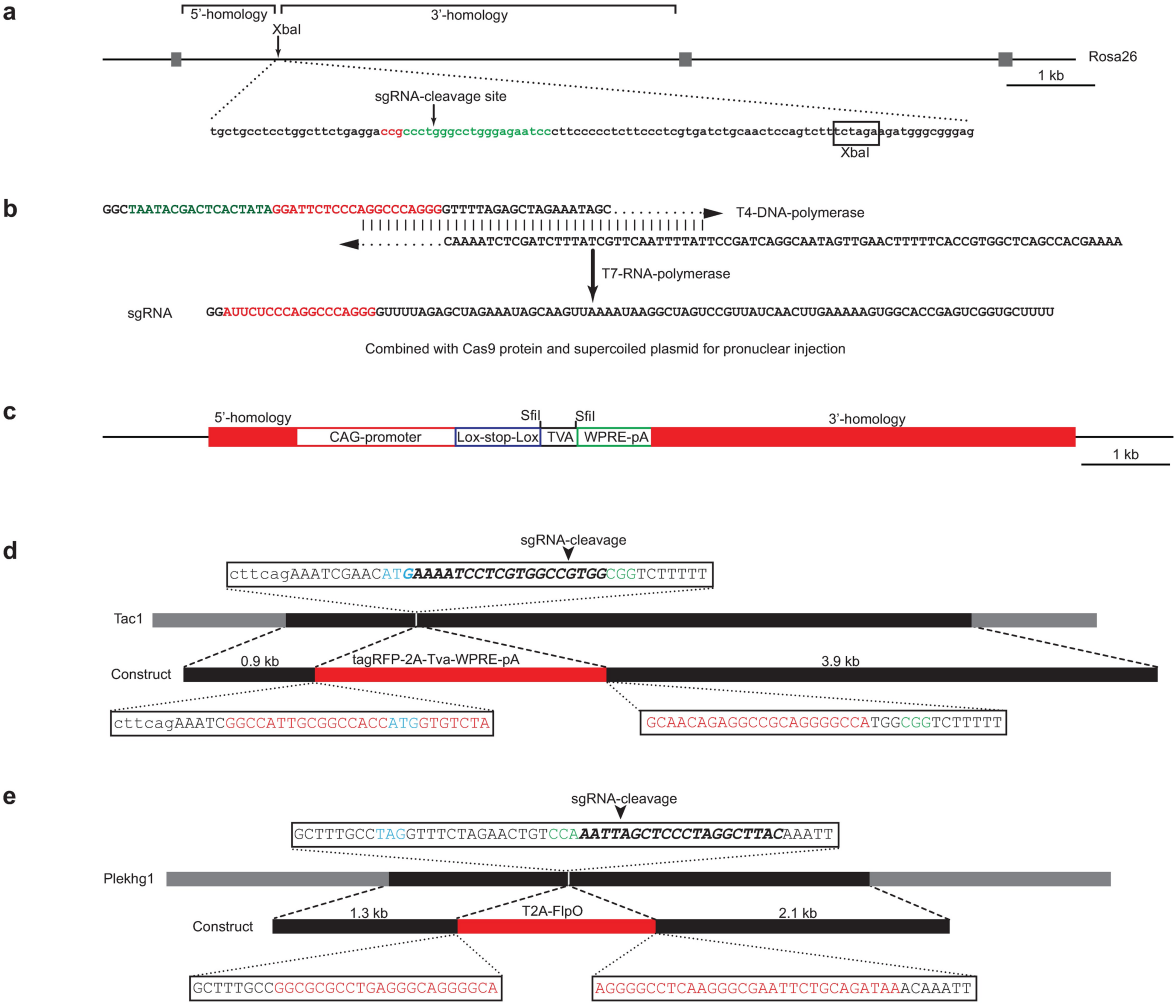
Strategy for CRISPR-directed knockin targeting *Rosa26 Tac1* and *Plekhg1* loci. (a) Schematic representation of the *Rosa26* locus showing the positions of the normal XbaI targeting site and the homology arms; below, the sequence around the XbaI site highlights the sgRNA used for CRISPR targeting. (b) The strategy used for CRISPR-mediated homologous recombination is highlighted for *Rosa26* targeting constructs. (c) The two *Rosa26* targeting constructs used a standard vector that was modified by deletion of the sgRNA recognition sequence, addition of a directional SfiI cloning site between the loxP-flanked transcriptional stop site and the woodchuck hepatitis virus posttranscriptional regulatory element (WPRE) and deletion of selection markers. Schematic representations of the strategy used for CRISPR directed generation of (d) the *Tac1-tagRFP-2A-Tva* and (e) *Plekhg1-2a-FlpO* mice; the genes indicating homology regions used (black boxes) and the sequence of the sgRNA (italic, bold) and PAM-site (green) used to direct CRISPR cleavage shown above a diagram of the construct used for homologous recombination highlighting the fusion junctions between homology arms (black) and knockin-genes (red). Relevant starting ATGs (*Tac1*) and stop codon TAG (*Plekhg1*) are highlighted in blue.

Synthesis of sgRNA followed published methods [15]. Briefly 60 base oligo nucleotides containing a T7 or Sp6 promoter sequence, the 20-base (or 21-base modified) target site (without the PAM site) and a 3’-complementary sequence necessary for generating the invariant region of sgRNAs were annealed to an 80-base constant oligo (see S1 Table). Single strand overhangs were filled in by T4-DNA-polymerase treatment to generate template for sgRNA synthesis (see Fig 1 and S1 Table). sgRNA synthesis used Megascript kits from Ambion or Ribomax kits from Promega according to the manufacturer's instructions. From a 50 μl reaction containing 100 pmol template DNA, T7-polymerase typically generated 150 μg sgRNA and Sp6 polymerase 30 μg sgRNA. All sgRNAs were tested *in vitro* using Cas9 (New England Biolabs) and a suitable PCR amplified or plasmid DNA target. sgRNAs were ethanol precipitated, resuspended at 1 mg/ml in water and stored at -80 °C. Immediately before use sgRNAs were heated to 80 °C for 5 minutes and cooled on ice to disrupt potential secondary structure.

### Vectors for homologous recombination

All vectors for homologous recombination were constructed using standard methods and lacked recognition sites for sgRNAs; coding sequences and all joins were confirmed by DNA-sequencing. Briefly homology arms for *Plekhg1* were generated from genomic DNA by PCR amplification incorporating appropriate cloning sites for plasmid construction (S1 Table). For *Tac1*, Bac-recombineering [16] was used to incorporate appropriate restriction sites at the starting ATG; an XbaI fragment containing the modified DNA was subcloned to a plasmid for construction of the targeting construct. *Rosa26* plasmids were generated from the AI9 plasmid [17], a gift from Hongkui Zeng (Addgene plasmid # 22799) by a series of steps including a restriction digest with PacI and cleavage using Rosa sgRNA1 directed Cas9 followed by recircularization to remove sgRNA recognition sequences. *tva* was amplified by PCR and compatible SfiI sites were used for directional cloning to generate the construct shown in Fig 1c. Extracellular-*Ruby2-sm-ollas* with a membrane anchor was generated using PCR to fuse the N-terminal signal peptide from mouse *Tas1r3* [18] to the non-fluorescent *mRuby2-sm-ollas* coding sequence [19] and a C-terminal transmembrane domain from *CD4* that has previously been used in GFP Reconstitution Across Synaptic Partners (GRASP) constructs [20]; again compatible SfiI sites allowed directional cloning in the Rosa26 targeting vector.

### Preparation of mixtures for oocyte injection

Oocyte injection components were mixed within 2 h of starting injection. Injections mixes used Cas9 protein (PNA Bio) and contained 100 ng/μl Cas9; 100 ng/μl sgRNA, 10 ng/μl plasmid DNA in 10 mM Tris pH7.5, 0.1 mM EDTA, 100mM NaCl, 30 μM spermine, 70 μM spermidine. Equal volumes of Cas9 protein and sgRNA (both 1 mg/ml) were mixed and incubated at 37 °C for 5 min; this complex was then diluted 5-fold with plasmid DNA and injection buffer and kept at room temperature till used for injection. Oocytes were incubated in medium containing 1 μM SCR7 (Xcessbio), a non-homologous end joining inhibitor [21] before and during pronuclear injection; no systematic tests were performed to determine SCR7 incubation increased targeted recombination frequency. Several injection mixes were tested for *in vitro* cleavage activity after completing pronuclear injection. We found no evidence for loss of activity of the Cas9-sgRNA complexes even after incubation at room temperature overnight.

### Animals, oocyte injections, viral transduction and expression analysis

C57BL/6J mice were used for all oocyte injection studies and crosses. Standard oocyte injection protocols [7] were used for generation of CRISPR assisted targeted mutants in the Transgenic Core Facility of National Institute Mental Health, NIH. Founders were identified using nested PCR-amplification with diagnostic primers for both 5’ and 3’ recombination.

For experiments to characterize engineered lines, mice aged 6–15 weeks of both genders were used. AAV constructs used were AAV1.*hSyn*.*Cre*.*WPRE*.*hGH* and AAV1.*hSyn*.*eGFP-Cre*.*WPRE*.*SV40* (Penn Vector Core); AAV-*hSyn-Coff/Fon EYFP-WPRE* (UNC Vector Core). EnvA pseudotyped rabies viruses (EnvA G-deleted Rabies-*mCherry*) was from the Salk Vector Core. Standard stereotaxic injections [22] were used to deliver viruses to appropriate brain regions; for injection mice were anesthetized using ketamine / xylazine and were provided with ketoprofen analgesia for 3 days post-surgery.

For visualization of expression of fluorescent proteins, tissue was fixed overnight using 4% paraformaldehyde in PBS; 16 μm frozen sections of appropriate brain regions and cranial ganglia were cut and collected on silanized slides. The ollas epitope was detected by immunostaining using the well characterized rat monoclonal L2 (MA5-16125, ThermoFisher) at a 1:50 dilution and visualized using a 1:200 dilution of donkey anti-rat IgG labeled with Alexa-594 (A-21209, ThermoFisher) secondary antibody. Tac1-expression was detected using a rabbit anti Substance P polyclonal (9450–0004, Biorad) at 1:50 dilution followed by a 1:200 dilution of donkey anti-rabbit IgG labeled with Alexa-488 (A-21206, Thermofisher). Trigeminal ganglion neurons were dissociated using enzymatic digestion with pronase and trituration. *In situ* hybridization used fresh-frozen sections and was performed using an RNAscope Multiplex Fluorescent Assay (Advanced Cell Diagnostics) as instructed by the manufacturer. Microscopy was with a Nikon C2 confocal; maximum intensity projections were exported to Adobe Photoshop for generation of composite images.

## Results and discussion

### Efficient generation of new lines of targeted mice

Over the past 25 years custom Bac-transgenic and knockin mice have revolutionized our understanding of many complex biological processes. CRISPR-Cas9 mediated homologous recombination appears likely to dominate the rapid generation of new complex knockin lines, and make generation of specialized animal models simpler, faster and more adaptable for specific needs. Here, we have used CRISPR-mediated homologous recombination in mouse zygotes to engineer new lines that may be generally useful. Four of these lines have been bred to homozygosity and are characterized here. Two involved targeting the *Rosa26* locus (see Fig 1), whereas the other two modified the *Tac1* and *Plekhg1* genes. The frequency of mice carrying correctly targeted alleles for these 4 lines was approx. 16% (13–19%; 14 out of 86 mice). PCR based screening revealed that few mice carried simple transgenic insertions of the targeting plasmids < 5% detected. The same number (4 of 86) carried incorrectly targeted alleles; these appeared to represent homologous recombination at the 5’ or 3’-end with some type of non-homologous end joining at the other end.

The chosen sgRNAs used here were predicted to be rather selective with at least one or two mutations in the 3’ region of the closest related sites. Therefore, given many reports suggesting only very low frequency modification of the predicted off-target sequences [7, 8, 21], we did not screen for additional CRISPR-dependent mutations; it should be noted that the lines can be backcrossed against wild type animals to further reduce the chance that mutations from off-target CRISPR modification might affect the results from the knockin alleles. All founder mice were backcrossed to C57BL/6 mice. In every case we observed that approximately 50% of offspring carried the targeted allele and none carried ectopic insertions of the targeting construct that were transmitted to offspring as expected for heterozygous knockin mice.

### Preliminary characterization of CRISPR-generated knockin mice

**Fig 2:**
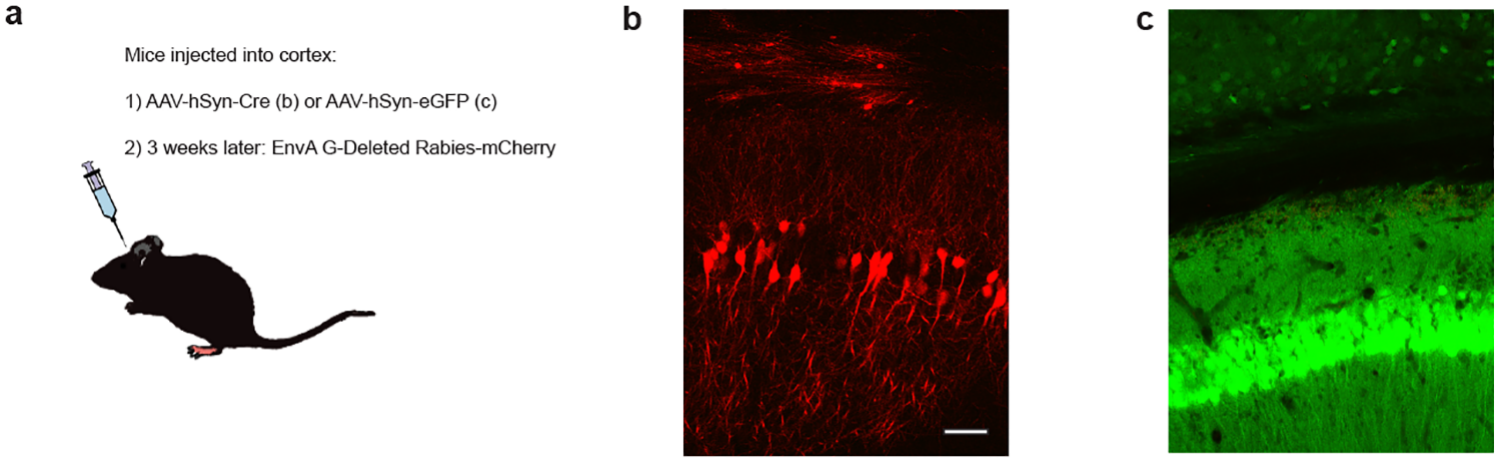
Characterization of *Rosa26-CAG-lox-stop-lox-TVA* mice. (a) Strategy and (b, c) results of testing heterozygous *Rosa26-CAG-lox-Stop-lox-TVA* mice for Cre-dependent expression of TVA. (b, c) Confocal images (combined red and green channels) of sections through the region of the brain injected with (b) AAV-hSyn-Cre or (c) AAV-hSyn-eGFP followed in each case by EnvA G-depleted Rabies-mCherry demonstrate that after Cre-recombination (b) EnvA-pseudotyped Rabies infected neurons in the forebrain but, (c) without Cre-expression no infection was observed; scale-bar 100 μm.

*Rosa26-CAG-lox-stop-lox-Tva* mice (Fig 2) were designed to allow Cre-dependent expression of the tva receptor for the avian sarcoma leucosis virus (ASLV) and thus to permit infection of Cre-expressing cells with viruses that have been pseudotyped with the envelope protein, EnvA from ASLV [23]. For example, such EnvA-pseudotyped rabies viruses have been used in retrograde circuit tracing experiments. Most often tva and the rabies glycoprotein (G) are supplied by transduction of Cre-expressing cells using adeno associated viral (AAV) constructs [23]. Indeed, in such studies, tva expressing neurons appear to be very efficiently infected even by extremely low viral titers of pseudotyped rabies virus. This new line should be useful for neural tracing studies [24] and also as a general means to direct infection of pseudotyped virus to Cre-expressing cells without AAV injection. Furthermore, we reasoned that since rabies rapidly kills infected neurons, it also provides a potential approach for targeted ablation of genetically and spatially defined populations of neurons. Finally, the recent development of new rabies variants with lower neuronal-toxicity [25] may make this line relevant for a much wider variety of functional studies.

To test and confirm that this line expresses functional tva in a Cre-dependent manner, we injected the cortex of heterozygous mice with an AAV expressing Cre-recombinase under the control of the neural human synapsin (*hSyn*) promoter; as a control, knockin animals were instead transduced with AAV-*hSyn-GFP*. Three weeks after AAV injection, EnvA-pseudotyped rabies designed to express mCherry was injected to the same region of the forebrain. After 7 days animals were euthanized and sections through the injection site were imaged for expression of fluorescent markers (Fig 2b and 2c). A clear difference in mCherry fluorescence in experimental and control animals demonstrates that this line expresses tva in a Cre-dependent manner.

**Fig 3:**
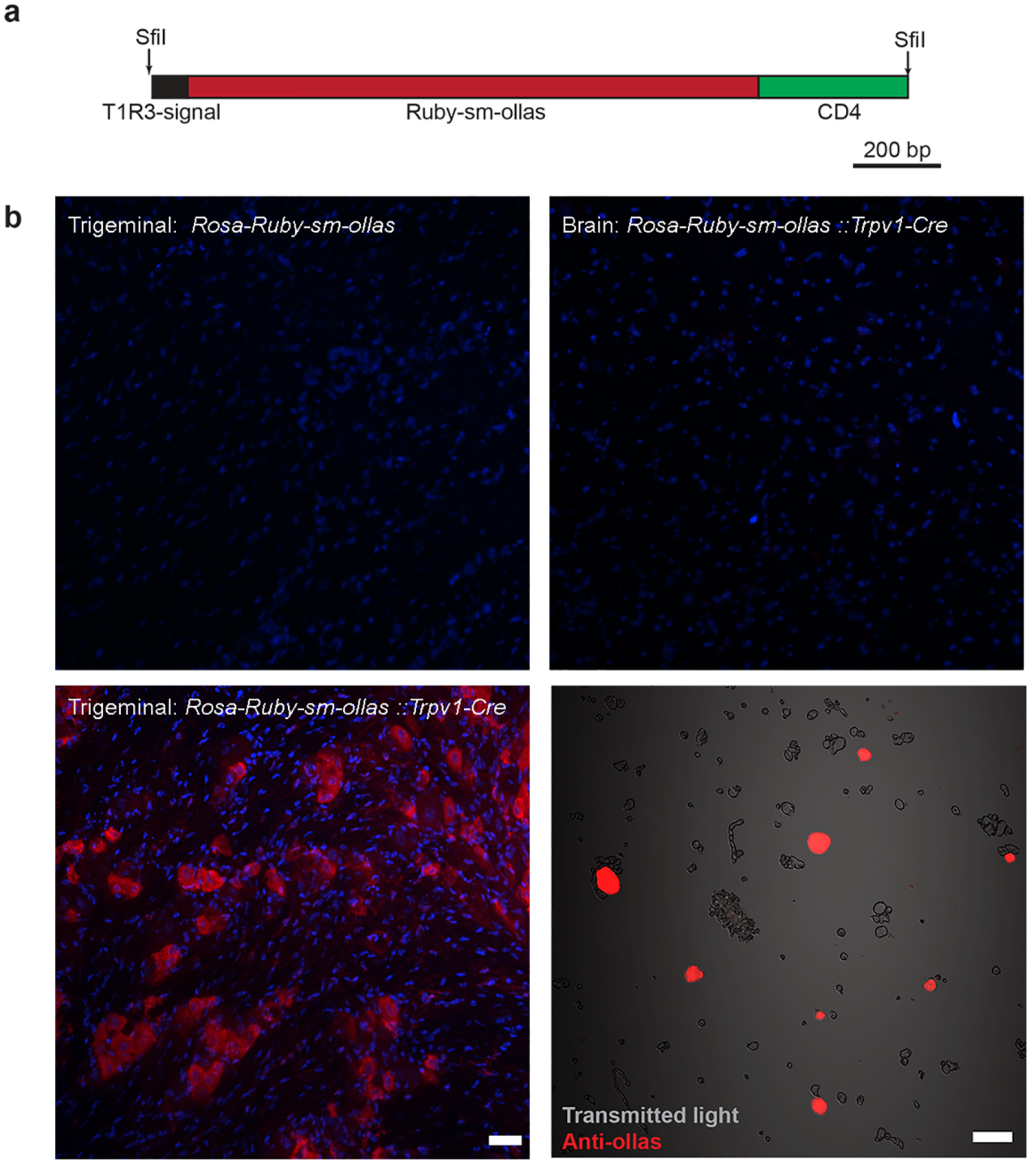
Characterization of *Rosa26-CAG-lox-stop-lox-extracellular-Ruby2-sm-ollas* mice. (a) Schematic representation of the synthetic construct used for cell surface expression of the ollas antigen: the mouse *Tas1R3* signal peptide was attached to a non-fluorescent *mRuby-ollas* spaghetti monster that was fused to the *CD4* transmembrane domain. (b) Confocal images of tissue sections and cells immune-stained using anti-ollas immunofluorescence (red); tissue sections were counterstained with DAPI (blue). Note that as expected, no expression of the ollas-tagged mRuby was detected in the trigeminal ganglion of *Rosa-CAG-lox-stop-lox-extracellular-ollas*-tagged mice in the absence of a *Cre*-transgene (upper-left panel) or in the brain of the knockin crossed into a *Trpv1-Cre* background (upper-right panel). In contrast, the trigeminal ganglia of these double positive mice (lower-left panel) prominently express mRuby-ollas; note that many small and medium diameter cells express the ollas-tag, scale-bar = 50 μm. Lower-right panel, trigeminal ganglion cells were isolated by enzymatic digestion and were stained without permeabilization; positive cells (red) can be distinguished from debris and non-labelled cells in the superimposed transmitted light image (gray), scale bar = 50 μm.

*Rosa26-CAG-lox-stop-lox-extracellular-Ruby2-sm-OLLAS* mice were designed to target expression of a cell-surface form of a non-fluorescent Ruby2-spaghetti-monster [19] carrying many copies of the “OLLAS”-antigen in a Cre-recombinase dependent manner. This is analogous to the approach taken to generate a conditional tva-line (Fig 2) and a simple extension of well reported strategies for generating Cre-reporter lines [17]. As indicated (Fig 3a), Ruby2-sm-OLLAS was modified to include an N-terminal signal-peptide [18] and a C-terminal membrane anchor [20]. We envisage that these mice will be valuable for selective isolation of cell-populations using panning and magnetic-bead approaches to quickly and gently enrich or deplete Cre-expressing cells.

As anticipated, the conditional *Ruby-sm-ollas* mice did not express mRuby-sm-ollas in the absence of Cre-recombination (Fig 3b, upper-left panel). Note that native fluorescence would not be observed because the mRuby encoded by this construct contains a (Gly)_3_ mutation at the chromophore site [19] therefore we screened for ollas-expression using a well characterized anti-ollas rat monoclonal, L2. To test for mRuby-sm-ollas expression after Cre excision, we crossed the condition *Ruby-sm-OLLAS* allele into a *Trpv1-Cre* background [26]. Previous reports demonstrate that in this bac-transgenic line, Cre-recombination occurs in sensory neurons in the trigeminal, dorsal root and vagal ganglia but not in the brain [26]. Because of developmental expression of Cre in this line, positive cells in the trigeminal ganglion include *Trpv1*-expressing neurons but are not limited to this cellular population in adult mice [26]. We used anti-ollas immunohistochemistry to examine expression of mRuby-sm-ollas in the double positive mice. As expected based on previous reports using this Cre-driver line [26], sections through the brain revealed no-expression of mRuby-sm-ollas (Fig 3b, upper-right panel). However, immunostaining of sections through the trigeminal ganglion demonstrated strong immune-detection of the ollas-epitope restricted to an appropriate subset of cells in the trigeminal ganglion (Fig 3, lower-left panel). To ensure that the epitope tag could also be detected in intact cells, we isolated neurons using papain and gentle trituration. An appropriate subset of this neural preparation was efficiently labeled using a rat monoclonal antibody against the epitope tag (Fig 3, lower-right panel) confirming surface expression of the modified mRuby2 and its resistance to papain cleavage.

*Tac1* is widely expressed in neurons in various regions of the brain including several involved in sensory processing and also in the sensory ganglia. In previous studies using *Tac1-CreER* mice [27] to attempt to label these cells, a major problem has been animal to animal variation in Cre-mediated recombination. The *Tac1-tagRFP-2A-TVA* mice generated in this study target *tagRFP* to the starting ATG of the *Tac1*-gene (Fig 1d). We hoped this knockin line would provide a more reliable approach to label and target *Tac1*-expressing neurons and/or help explain the variable expression pattern observed in the *Tac1-CreER* animals. It should be noted that homozygous knockin mice are also *Tac1* knockout animals.

**Fig 4:**
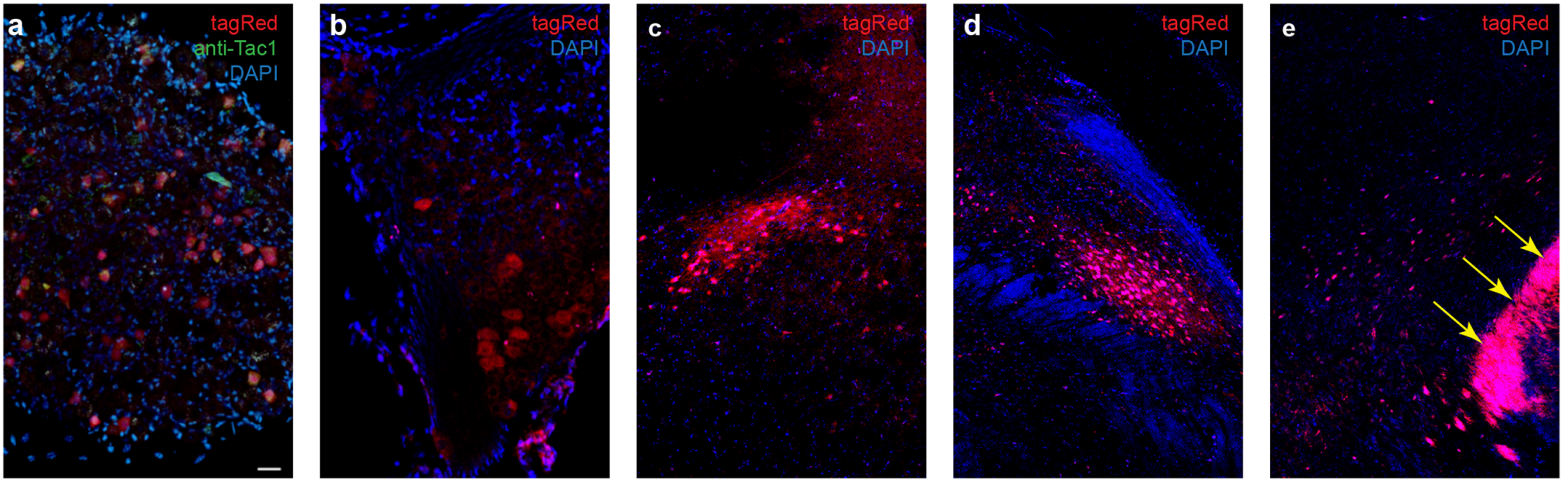
Characterization of *Tac1-tagRFP-2A-Tva* mice. Fluorescence images of sections of sensory ganglia and regions of the brain illustrate the cellular expression of tagRFP (red). (a) Representative section of the trigeminal ganglion, immunostained for Tac1 (green) and counter-stained with DAPI (blue) demonstrates that tagRFP (red) is expressed in the subset of trigeminal neurons that stain positive for Tac1. In (b) the geniculate ganglion, (c) the brainstem (nucleus of the solitary tract), (d) the parabrachial nucleus and (e) the para-subthalamic nucleus (PSTN), tagRFP (red) is also expressed in subsets of neurons. Note the strongly positive fiber tracts (arrowed) running close to the PSTN; scale bar = 100 μm.

*Tac1* is prominently expressed in a subset of somatosensory neurons, and, as expected, *Tac1-tagRFP-2A-Tva* mice reliably express tagRFP in trigeminal neurons (Fig 4a). We used immunostaining to confirm good correspondence of Tac1 and tagRFP expression (Fig 4a). In other sensory ganglia and several regions of the brain tagRFP expression was, as expected, also widely detected (see Fig 4b–4e) but we observed mouse to mouse variation in heterozygous animals, just as we had when using *Tac1-CreER* knockin lines. Together these results point to a complex control of *Tac1*-gene expression in the brain. Future experiments to understand the basis for this variation will likely be aided by the availability of a mouse line where Tac1-gene expression is marked by strong RFP-fluorescence.

**Fig 5:**
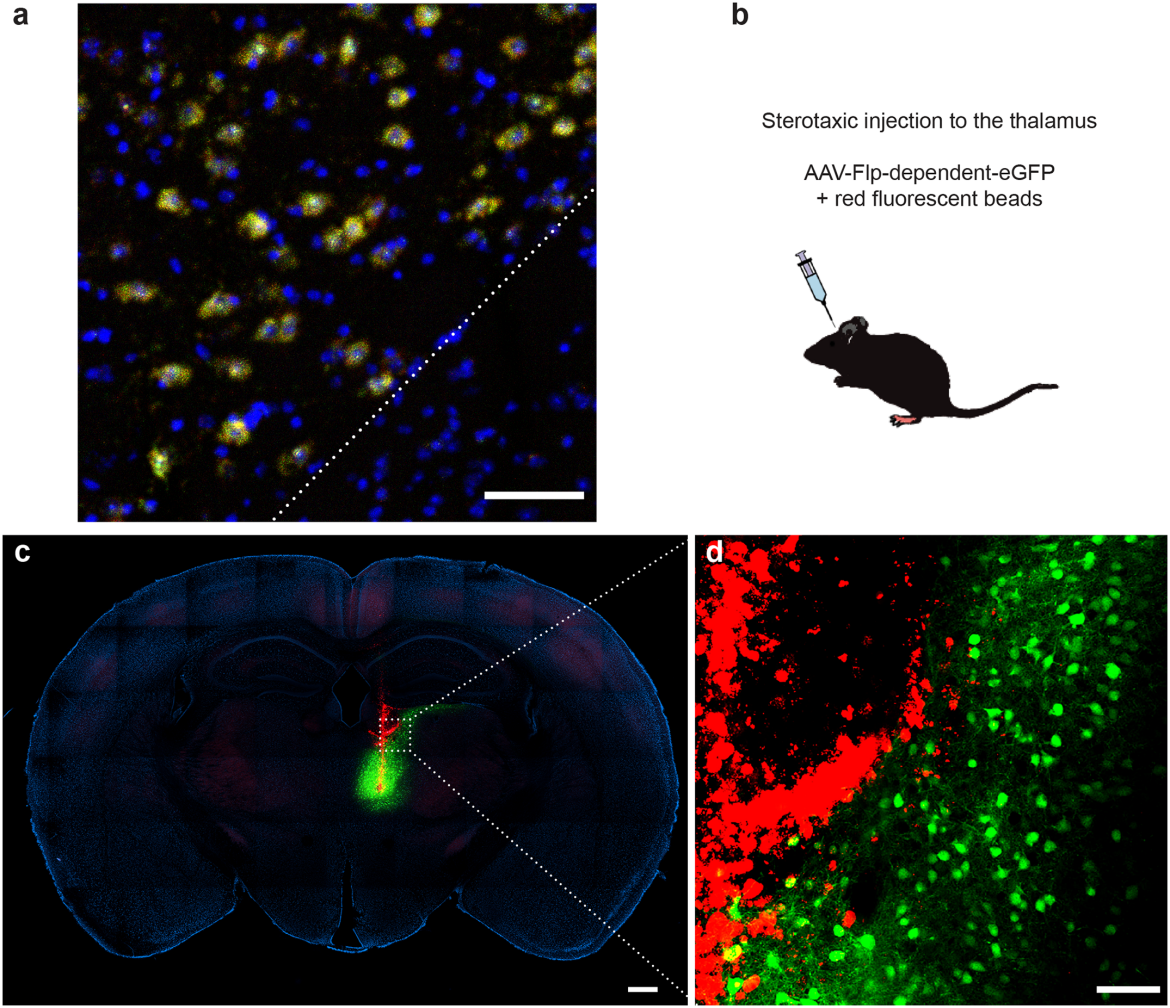
Characterization of *Plekhg1-2A-FlpO* mice. (a) Section through the thalamus of a *Plekhg1-2A-FlpO* mouse stained by in situ hybridization with probes for *Plekhg1* (green) and *FlpO* (red); nuclei are counterstained with DAPI (blue). Note that *Plekhg1* (and *FlpO*) expression is limited to a subset of cells in the thalamus; neighboring brain regions (e.g. below and to the right of the dotted line) do not express these transcripts. (b-d) Strategy characterizing *Plekhg1*-directed expression of FlpO-activity. Heterozygous mice were injected with a Flp dependent reporter virus as indicated schematically in (b). (c, d) Fluorescence images of a section through the thalamus of an injected mouse illustrates the injection tract (red) and FlpO dependent expression of eYFP (green) in (c) a stitched image of the entire section and (d) a magnified view of the boxed region to highlight cellular expression; scale bars (a) = 50 μm (c) = 500 μm, (d) = 100 μm.

The fourth line generated in this study, *Plekhg1-2a-FlpO* (Fig 5), was designed to allow expression of Flp-recombinase in Plekhg1-expressing cells. *Plekhg1* is a highly expressed marker of a large group of neurons in the thalamus (an important station in sensory circuits). It is also expressed in subsets of neurons in the dorsal root ganglion and dorsal horn as well as a range of non-neuronal cells. We were particularly keen to target FlpO to thalamic neurons to allow intersectional approaches whereby Cre recombinase could be expressed from a different promoter for targeting restricted subsets of these cells. Here we used double label *in situ* hybridization to confirm expression of *Plekhg1-2a-FlpO* in thalamic neurons but not adjacent brain regions (Fig 5a). We then used stereotaxic injection of Flp dependent AAV expressing a fluorescent protein (eYFP) into the thalamus to test and validate appropriate expression of functional FlpO. Red-fluorescent beads were co-injected with the AAV-construct to mark the site of injection. As shown (Fig 5b and 5c), the injection was localized to the dorsomedial thalamus with plenty of beads (and thus virus) lining the injection track. In contrast, eYFP-expression was limited to the thalamus with many positive neurons visible in higher magnification images (Fig 5c). This expression pattern closely matches the pattern of *Plekhg1*-expression in this region of the brain validating the line as a means of targeting the thalamus.

In summary, here we describe the generation and characterization of new lines of knockin mice engineered using CRISPR directed homologous recombination. As expected, use of CRISPR makes generation of complex knockin mice at these three loci as efficient and simple as generation of standard transgenic lines. We anticipate that the four lines described and characterized here will each serve as a useful resource; we will make the mice available to the community as frozen sperm.

## Supporting information

S1 Fig Specificity and efficiency of sgRNAs tested prior to generation of mice. a) Upper-panel schematic representation of test plasmid for digestion with *Rosa*-directed sgRNA-Cas9; an AI9 derived plasmid (~16,600 bp) was digested with SfiI and XhoI to yield 3 fragments (~8, 7 and 1.5 kb). Digested plasmid DNA was incubated with Cas9 (NEB) or Cas9 premixed with excess Rosa-directed sgRNA according to the manufacturer’s instructions (lower panel). In the presence of the sgRNA the 7 kb band was cleaved at the expected site to yield fragments of 4 and 3 kb. (b) sgRNAs directed to cleave *Tac1* and *Plekhg1* were tested for cutting efficacy and specificity using PCR-products from mouse genomic DNA cloned into pCR8/GW/TOPO (Thermo Fisher; see upper panel). The two plasmids were linearized with XhoI and DNA were incubated with Cas9 mixed with sgRNA as indicated in the lower panel. The *Tac1* vector was efficiently cut by *Tac1*-sgRNA Cas9 to yield two equal sized bands that migrate at about 1.8 kb; similarly, the *Plekhg1* vector was cleaved by the appropriate sgRNA-enzyme mix. As expected neither sgRNA directs cutting of the inappropriate vector.
(TIF)

S1 Table List of oligonucleotides used to generate and screen knockin mice. Oligonucleotides used for generation of mice described in this study with details of use.
(XLSX)
