# Supplementary figures and images for: Characterization of knockin mice at the Rosa26, Tac1 and Plekhg1 loci generated by homologous recombination in oocytes
Source: PLoS One. 2018 Feb 27;13(2):e0193129. doi: 10.1371/journal.pone.0193129 (PMC5828354; doi:10.1371/journal.pone.0193129)

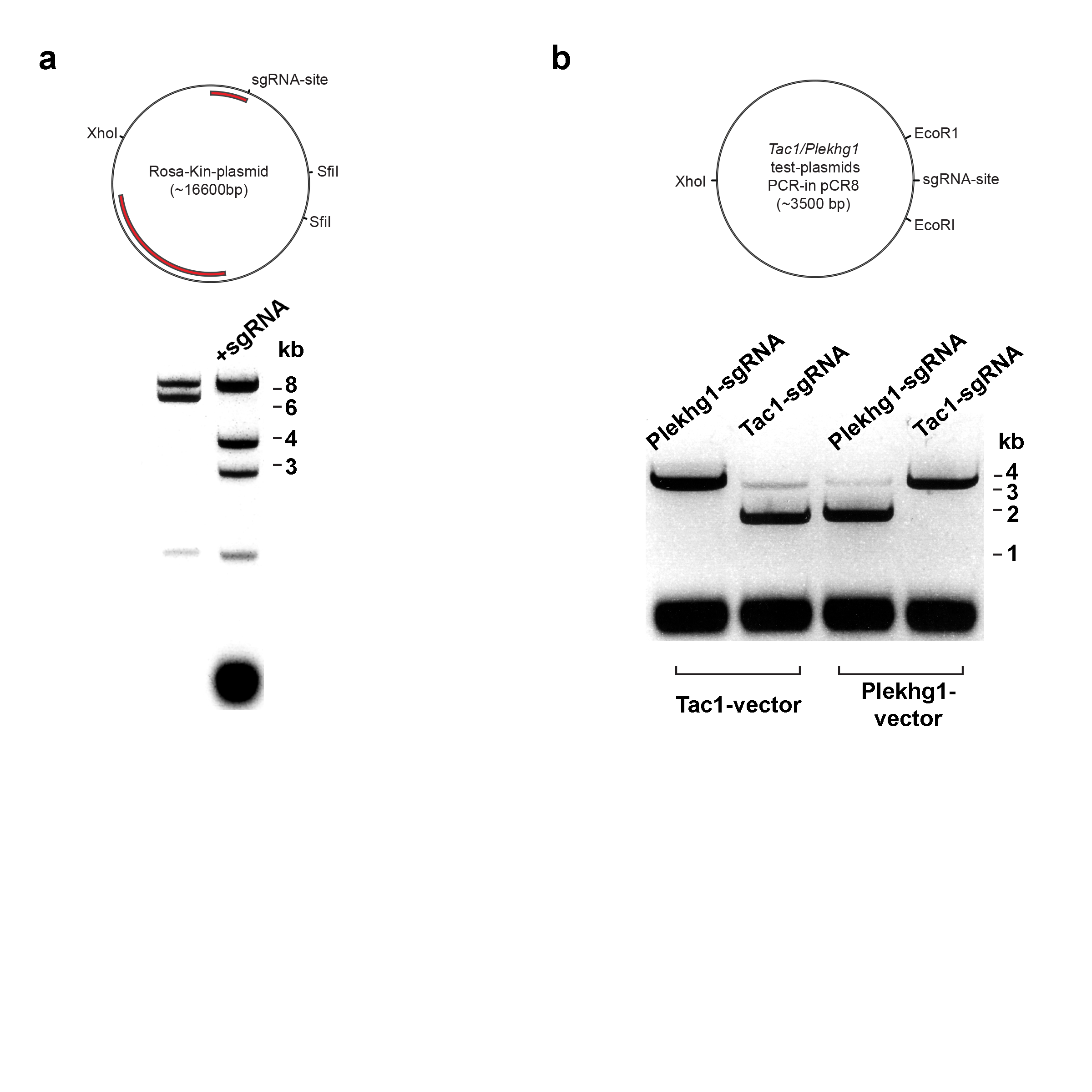

Supplement: S1 Fig — a) Upper-panel schematic representation of test plasmid for digestion with Rosa-directed sgRNA-Cas9; an AI9 derived plasmid (~16,600 bp) was digested with SfiI and XhoI to yield 3 fragments (~8, 7 and 1.5 kb). Digested plasmid DNA was incubated with Cas9 (NEB) or Cas9 premixed with excess Rosa-directed sgRNA according to the manufacturer’s instructions (lower panel). In the presence of the sgRNA the 7 kb band was cleaved at the expected site to yield fragments of 4 and 3 kb. (b) sgRNAs directed to cleave Tac1 and Plekhg1 were tested for cutting efficacy and specificity using PCR-products from mouse genomic DNA cloned into pCR8/GW/TOPO (Thermo Fisher; see upper panel). The two plasmids were linearized with XhoI and DNA were incubated with Cas9 mixed with sgRNA as indicated in the lower panel. The Tac1 vector was efficiently cut by Tac1-sgRNA Cas9 to yield two equal sized bands that migrate at about 1.8 kb; similarly, the Plekhg1 vector was cleaved by the appropriate sgRNA-enzyme mix. As expected neither sgRNA directs cutting of the inappropriate vector. (TIF) [file pone.0193129.s001.tif]
